# Imaging of Joints and Bones in Autoinflammation

**DOI:** 10.3390/jcm9124074

**Published:** 2020-12-17

**Authors:** Katharina Ziegeler, Iris Eshed, Torsten Diekhoff, Kay Geert Hermann

**Affiliations:** 1 Department of Radiology, Charité—Universitätsmedizin Berlin, 10117 Berlin, Germany; torsten.diekhoff@charite.de (T.D.); kgh@charite.de (K.G.H.); 2 Department of Diagnostic Imaging, Sheba Medical Center, Tel Giborim Affiliated with the Sackler School of Medicine, Tel Aviv University, 52621 Tel Aviv, Israel; iris.eshed@sheba.health.gov.il

**Keywords:** imaging, autoinflammation, arthritis

## Abstract

Autoinflammatory disorders are commonly characterized by seemingly unprovoked systemic inflammation mainly driven by cells and cytokines of the innate immune system. In many disorders on this spectrum, joint and bone involvement may be observed and imaging of these manifestations can provide essential diagnostic information. This review aimed to provide a comprehensive overview of the imaging characteristics for major diseases and disease groups on the autoinflammatory spectrum, including familial Mediterranean fever (FMF), Behçet disease (BD), crystal deposition diseases (including gout), adult-onset Still’s disease (AoSD), and syndromatic synovitis, acne, pustulosis, hyperostosis, and osteitis (SAPHO)/chronic recurrent multifocal osteomyelitis (CRMO). Herein, we discuss common and distinguishing imaging characteristics, phenotypical overlaps with related diseases, and promising fields of future research.

## 1. Introduction

Autoinflammatory disorders, in contrast to classical autoimmune disorders, are commonly characterized by seemingly unprovoked systemic inflammation without auto-reactive T-lymphocytes or auto-antibodies [1]. The inflammatory process is mainly driven by cells and cytokines of the innate immune system. During the past decade, the understanding of auto-inflammation and auto-immunity has shifted away from a concept of two distinct groups of disorders towards a spectrum of disorders [1,2]. Although joint involvement in varying degrees may be observed in many autoinflammatory diseases, there are a number of diseases within this spectrum where imaging has special significance in the diagnostic process, i.e., familial Mediterranean fever (FMF), Behçet disease (BD), crystal deposition diseases, adult-onset Still’s disease (AOSD), and syndromic synovitis, acne, pustulosis, hyperostosis, and osteitis (SAPHO)/chronic recurrent multifocal osteomyelitis (CRMO). The aim of this article was to provide an overview of the state of the art joint imaging techniques in these disease groups, and to point out promising fields of future research.

## 2. Familial Mediterranean Fever

Familial Mediterranean fever (FMF) is an autoinflammatory autosomal recessive disorder that usually begins before the age of 20 and causes recurrent fever and serosal inflammation of the abdomen, lungs, and joints, leading to severe pain [3]. FMF is commonly seen in people of Mediterranean and Middle Eastern descent, including Jews, Armenians, Arabs, Kurds, Greeks, Turks, Iranians, and Italians. It is caused by mutations in the Mediterranean fever (MEFV) gene, the product of which, the pyrin protein, is involved in the control of inflammation [4].

Arthralgia of the large joints of lower extremities including hip, knee, or ankle joints is common. The patient often presents with severe pain in one joint. Very rarely, multiple joints are affected simultaneously. The pattern of involvement of a large, lower extremity joint conjures a clinical resemblance to spondyloarthropathy (SpA). Indeed, the incidence of SpA in FMF patients was reported to be up to 7% of the total patient population. Moreover, up to 27% of patients with sacroiliitis had joint involvement [5] and a significantly higher frequency of M694V. Nonetheless, these patients maintained low HLA-B27 positivity [6].

**Figure 1:**
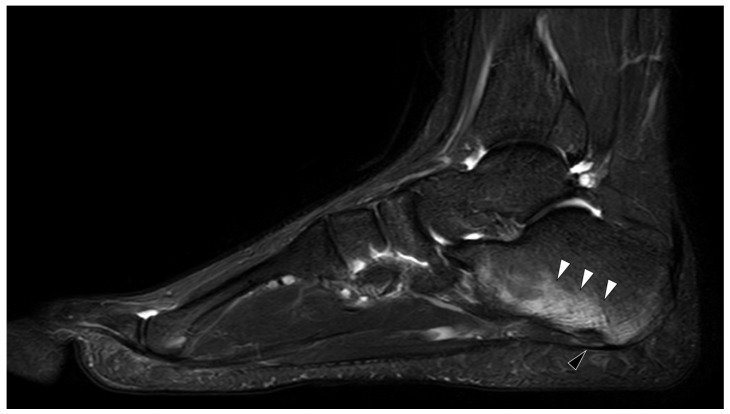
MRI in Familial Mediterranean fever (FMF). Sagittal T2 weighted with fat saturation image of an ankle of an 18 years old male with known FMF and exertional leg pain. There is characteristic enthesitis (black arrowhead) with extensive calcaneal bone marrow edema (white arrow heads) at the insertion of the long plantar tendon.

Enthesitis, which is the hallmark of SpA, was also reported in FMF, mainly in the calcaneal insertion of the Achilles tendon, the plantar fascia, and/or the long plantar ligament [7]. The characteristic MRI features of this ankle enthesitis reported in SpA are insertional bone marrow edema (BME), thickening and high signal intensity of the affected tendon, and increased synovial fluid in the adjacent bursa [8]. A unique MRI feature in FMF is significant calcaneal BME along the insertion site of the long plantar tendon—an imaging example is given in Figure 1 [9]. This ankle enthesopathy of FMF patients is related to exertional leg pain that is a common debilitating symptom of FMF.

## 3. Behçet Disease

Behçet disease (BD) is an auto-inflammatory systemic vasculitis of unknown etiology. BD is characterized by mucocutaneous manifestations (i.e., recurrent oral and genital ulcerations), ocular manifestations (especially chronic relapsing uveitis and systemic vasculitis involving arteries and veins of all sizes), and peripheral arthritis [10]. Although BD does not follow a Mendelian inheritance, it is associated with HLA-B51/B5, and carriers are at high risk of developing BD compared to non-carriers [11].

Arthritic manifestation is one of the minor manifestations and it is usually overlooked. Joint involvement is typically non-erosive and non-deforming arthritis, seen in 50% of BD patients [12,13]. The most commonly involved joints include the knees, ankles, elbows, wrists, fingers, and toes [13,14]. Erosive forms of arthritis in BD are uncommon, and the most affected locations are the axial joint (sacroiliac), enthesis (calcaneal), and peripheral joints, such as metatarsophalangeal and interphalangeal joints of the feet [15]. Repeated attacks of synovitis in the same joint leads to a destructive arthritis resembling the radiological changes of rheumatoid arthritis. There are various variable reports on the prevalence of sacroiliitis and enthesitis in BD. While some report high prevalence, others claim that there is only rare involvement [15,16,17].

The coexistence of BD and SpA, as well as the presence of clinical overlap between BD and some SpA subgroups (i.e., inflammatory bowel disease and reactive disease) suggest a potential common pathogenesis. However, this has not yet been proven.

## 4. Crystal Deposition

In terms of prevalence, crystal-induced arthritides are the most common diseases on the autoinflammatory spectrum [18]. The establishment of their inflammatory nature dates back less than 20 years [19,20]. Since then, the capacity of both mono-sodium urate (MSU) and calcium species to activate the NLRP3 inflammasome [21], as well as the production and secretion of pro-inflammatory cytokines, has been widely accepted [22,23]. To date, the gold standard for diagnosis remains the demonstration of crystals in synovial fluid [24,25]. As joint aspiration is an invasive procedure, the need for improved diagnostic imaging is well established. Over the last few years, a number of imaging studies have greatly advanced the detection of MSU, calcium pyrophosphate (CPP), and basic calcium species (BCP). A common denominator of all crystal deposition diseases, however, is the fact that deposition on imaging should not be equated with disease. For CPP, community-based cross-sectional studies estimate the prevalence of deposition between 7.0% [26] and 8.1% [27], while estimates of symptomatic disease are well below 1% of the general population [28]. Asymptomatic hyperuricemia is estimated to affect approximately 2.6% of the general population [29], while the prevalence of symptomatic gout lies much lower, between 0.46% [28] and 1.1% [29]. Therefore, imaging of crystal deposition disease poses unique challenges, which are addressed in the following paragraphs.

## 5. Gout

**Figure 2:**
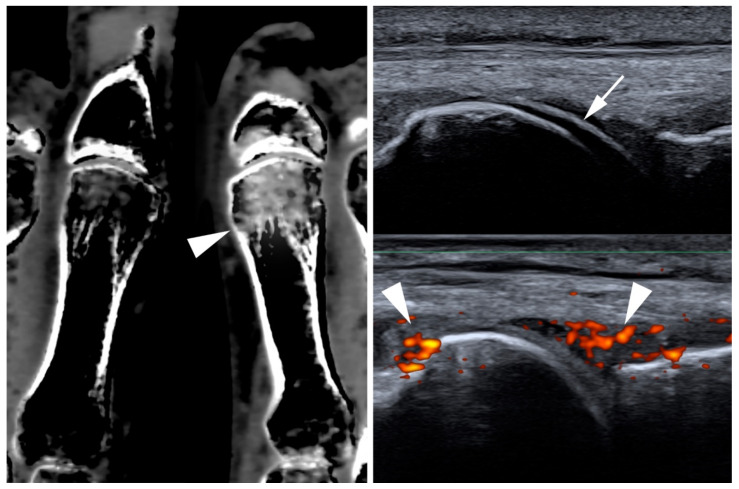
Multimodality imaging for gout. (**Left**): Virtual calcium subtraction imaging from dual-energy computed tomography. The arrowhead indicates bone marrow edema in the first metacarpal head. (**Right**): Ultrasound image of the same patient. The arrow indicates double-contour sign and arrowheads indicate synovitis on the power Doppler.

Historically, radiography has been the main imaging modality for investigating gout [30]. However, as a radiograph is only able to reliably capture advanced stages of the disease, recent years have seen a shift towards cross-sectional imaging techniques. One of the most available, inexpensive, and non-invasive imaging techniques in point-of-care rheumatology is the ultrasound. Using ultrasounds, MSU depositions may be demonstrated in tendons, periarticular soft tissue, and articular cartilage (i.e., the double-contour sign) [31] with high sensitivity and specificity [32,33]. Longitudinal studies have also demonstrated the capacity of ultrasound to monitor diseases [34]. Additionally, ultrasounds can visualize erosions, joint effusion, and synovitis as surrogates of inflammation [35]. Dual-energy computed tomography (DECT) has become a well-established tool in gout imaging and was included in the 2018 update of the American College of Rheumatology (ACR) and European League Against Rheumatism (EULAR) classification criteria [24]. Its specificity and sensitivity have estimated to be 93.6% and 84.7% in a recent meta-analysis [36], yet its diagnostic accuracy may be lower in cases of recent onset gout [37,38]. Apart from establishing the diagnosis, DECT can be used as a tool for quantification of urate burden [39]. As such, it may be applied as a surveillance tool in urate lowering therapy [40]. Additionally, there is evidence that DECT may be useful to depict bone marrow edema, allowing for a more direct visualization of acute inflammation [41]. Clinical imaging examples of gout are supplied in Figure 2.

## 6. Calcium Pyrophosphate Dihydrate Deposition (CPPD)

**Figure 3:**
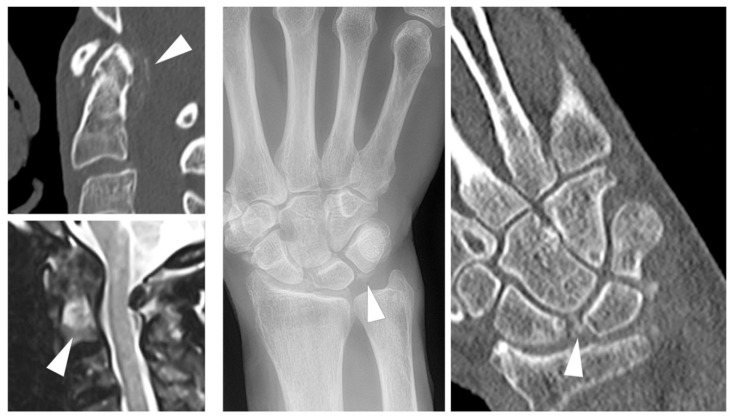
Multimodality imaging in Calcium Pyrophosphate Dihydrate Deposition (CPPD). (**Left**): Crowned dens syndrome with flake-like calcifications in the CT image (arrowhead) and concurring bone marrow edema on MRI (arrowhead). (**Right**): CPPD of the wrist, showing calcifications of the luno-triquetral ligament on radiography and additional calcifications of the scapho-lunate ligament on CT (arrowhead).

The most widely applied and accepted imaging modality for the diagnosis of CPPD remains radiography [25], where linear or flake-like calcifications in typical localizations (e.g., the hyaline cartilage of the knee, or the triangular fibrocartilage of the wrist) may be demonstrated. Nevertheless, CPPD imaging has seen a steady advance in cross-sectional imaging techniques in recent years. Already embedded in the 2015 EULAR recommendations for the diagnosis of CPPD [25], ultrasonography has gained increased attention in recent years. This has been facilitated by the establishment and preliminary validation of ultrasonographic criteria for CPPD by the dedicated Outcome Measures in Rheumatology (OMERACT) taskforce [42,43]. A major strength of ultrasonography in CPPD imaging is its capacity to visualize inflammation by demonstration of synovitis using a power Doppler [35]. Computed tomography (CT) has long been established as an imaging tool in CPPD manifestations at the axial skeleton, especially the atlanto-axial joint (crowned dens syndrome) [44], but recent studies have applied it to the wrist [45] and knee [46], thus putting a new focus on crystal depositions, not only in cartilage but also in ligaments. The use of DECT in the diagnosis of CPPD remains controversial. Although in vitro and in vivo studies show an encouragingly high capacity for differentiation between different calcium species [47,48,49], evidence of added diagnostic value of DECT vs. conventional CT remains sparse [50,51]. However, DECT may be a valuable tool for strengthening the understanding of the development of specific patterns of arthropathy in CPPD as it can be used to non-invasively detect tissue remodeling [52]. To date, evidence of the usefulness of MRI in CPPD imaging is sparse. In spinal imaging, MRI may be useful for assessing acute inflammation when CPP deposition is established using alternative imaging, such as CT [53]. Imaging examples from different modalities are given in Figure 3.

## 7. BCP and Mixed Crystal Disease

**Figure 4:**
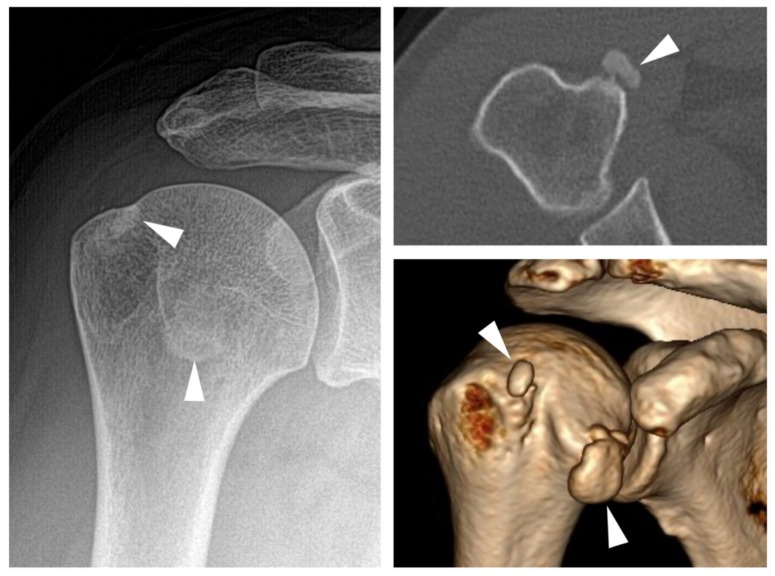
BCP deposition. (**Left**): White arrowheads indicate calcific deposition on radiography. (**Right**): Axial and 3D reconstructions of the same shoulder with better visualization of the depositions.

Basic calcium deposition (BCP) comprises a heterogeneous spectrum of conditions associated with a number of different calcium containing crystal species, the most common of which is hydroxy-apatite deposition disease (HADD) [54]. In terms of imaging characteristics, BCP may be distinguished from CPP crystal deposition, both by localization and calcification morphology. While HADD typically manifests as circumscribed calcific deposits inside of tendons, especially at the tendons of the rotator cuff [55], CPP crystals are typically found in ligaments and hyaline or fibrocartilage as ill defined, flake-like depositions [56]. An example of a symptomatic BCP deposit is provided in Figure 4. The most commonly applied imaging modality is radiography, which is usually sufficient for visualizing these depositions. Identification of calcium deposition on MRI imaging can be challenging, but three-dimensional imaging allows for the direct visualization of invasion of the deposit into the bursa or bone. The size of the calcific deposit does not correspond with the intensity of symptoms [57]. Symptom onset is typically observed when resorption of the calcification commences [58]. In this phase, macrophages invade [59] and, as a result, local edema, redness, swelling, and tenderness may be observed. This can be accompanied by intense pain and decreased range of motion. During this phase, calcium crystals may enter the subacromial-subdeltoid bursa [55]. 

A special subtype of BCP is the Milwaukee (shoulder/knee) syndrome [60]. This rare arthropathy exhibits a rapidly progressive joint destruction, often affects older women, and is connected with rotator cuff tears [61]. Synovial fluid aspiration yields a mixture of calcium crystals (predominantly hydroxy-apatite) and sero-hematic synovial fluid with low leucocyte counts [62].

## 8. Adult-Onset Still’s Disease (AOSD) 

Still’s disease is a rare systemic auto-inflammatory disease that often poses a diagnostic challenge to clinicians. Among the clinical features of the disease are arthralgia and arthritis, which typically concur with classical fever spikes. Joint involvement is considered a common manifestation and may be observed in at least two-thirds of affected patients. It may present at any joint, including the axial skeleton [63]. Biopsy of the synovium typically reveals non-specific synovitis [64] and synovial fluid analysis shows high cellularity with neutrophil predominance [65]. Although the arthritis is non-destructive in the majority of patients, approximately 30% of patients may develop erosions. In these patients, bilateral destruction of the carpus, with subsequent carpal ankylosis in the absence of erosive changes at the metacarpophalangeal and proximal interphalangeal joints, may be a valuable imaging feature for the distinction from rheumatoid arthritis [64]. Additionally, destructive arthritis of the distal interphalangeal joints in younger patients may be observed [66].

## 9. SAPHO and CRMO 

**Figure 5:**
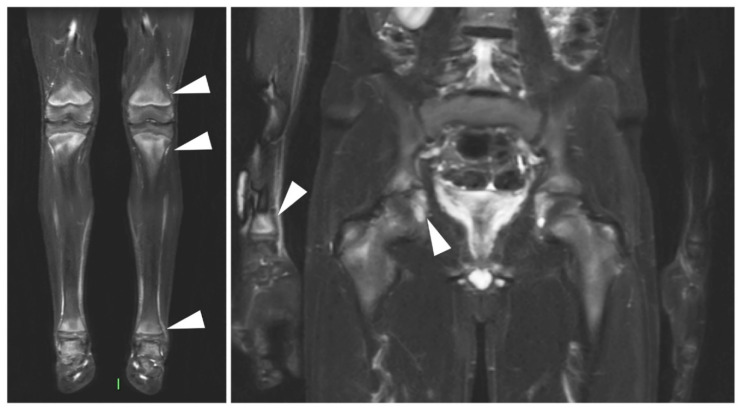
MRI in CRMO. Coronal T2 weighted with fat saturation images of a whole-body MRI in a 12 year old boy with chronic recurrent multifocal osteomyelitis (CRMO). There is evidence of bilateral bone marrow edema in the distal femur, distal/proximal tibia and talus, triradiate cartilage, and unilateral BME on the right distal radius (indicated by arrowheads. In the case of bilateral lesions, only one side was annotated).

The syndromes synovitis, acne, pustulosis, hyperostosis, and osteitis (SAPHO)/chronic recurrent multifocal osteomyelitis (CRMO) are considered related diseases, characterized mainly by neutrophilic inflammation, skin eruptions, and osteitis with bone hypertrophy [67]. Alternatively, the diseases are sometimes termed chronic non-bacterial osteomyelitis (CNO) [68]. The distribution of disease involvement differs in children and adults [69]. While the former typically presents with lesions in the long tubular bones and less frequently the spine and clavicles [70,71], the latter usually presents with involvement of the anterior chest wall, spine, and pelvis [72]. As many affected patients are children or adolescents, MRI is widely applied in the imaging of this disease family and may reliably depict osteitis in commonly affected sites [73]. An imaging example is supplied in Figure 5. However, radiography, and especially CT, are superior in the detection of hyperostosis and osteosclerosis, which are both well-established imaging characteristics of SAPHO/CRMO [74]. In adults with primary manifestations at the axial skeleton, differentiation from axial spondyloarthritis (axSpA) can be challenging; however, a valuable diagnostic clue is that generally sclerosis is more pronounced in patients with SAPHO/CRMO [69]. This imaging feature represents an interesting pathophysiological bridge towards the related axSpA spectrum. The predominantly auto-immune (e.g., B- and T-cell mediated) inflammation of the entheses in axSpA and psoriatic arthritis [75] shares many characteristics with the predominantly neutrophilic osteitis of SAPHO/CRMO [76].

## 10. Conclusions

Imaging is a vital tool to diagnose and follow-up on auto-inflammatory spectrum diseases. Although our knowledge of imaging features of specific auto-inflammatory diseases are steadily increasing, they remain particularly challenging to distinguish from auto-immune diseases in many cases, as cellular and cytokine profiles do not translate directly to imaging features. A better understanding of both common and distinguishing imaging features of auto-immune and auto-inflammatory diseases may increase our understanding of disease pathways in the future. 

## References

[1] McGonagle D., McDermott M.F. (2006). A Proposed Classification of the Immunological Diseases. PLoS Med..

[2] Hedrich C.M. (2016). Shaping the spectrum—From autoinflammation to autoimmunity. Clin. Immunol..

[3] Ozdogan H., Ugurlu S. (2019). Familial Mediterranean Fever. Press. Méd..

[4] Touitou I. (2001). The spectrum of Familial Mediterranean Fever (FMF) mutations. Eur. J. Hum. Genet..

[5] Kaşifoğlu T., Çalışır C., Cansu D.Ü., Korkmaz E.H.C. (2008). The frequency of sacroiliitis in familial Mediterranean fever and the role of HLA-B27 and MEFV mutations in the development of sacroiliitis. Clin. Rheumatol..

[6] Cosan F., Üstek D., Oku B., Duymaz-Tozkir J., Cakiris A., Abaci N., Ocal L., Aral O., Gul A. (2010). Association of familial Mediterranean fever-related MEFV variations with ankylosing spondylitis. Arthritis Rheum..

[7] Eshed I., Rosman Y., Livneh A., Kedem R., Langevitz P., Lidar M., Ben-Zvi I. (2014). Exertional Leg Pain in Familial Mediterranean Fever: A Manifestation of an Underlying Enthesopathy and a Marker of More Severe Disease. Arthritis Rheumatol..

[8] Eshed I., Bollow M., McGonagle D.G., Tan A.L., Althoff C., Asbach P., Hermann K.-G. (2007). MRI of enthesitis of the appendicular skeleton in spondyloarthritis. Ann. Rheum. Dis..

[9] Eshed I., Kushnir T., Livneh A., Langevitz P., Ben-Zvi I., Konen E., Lidar M. (2012). Exertional leg pain as a manifestation of occult spondyloarthropathy in familial Mediterranean fever: An MRI evaluation. Scand. J. Rheumatol..

[10] Feigenbaum A. (1956). Description of Behcet’s Syndrome in the Hippocratic Third Book of Endemic Diseases. Br. J. Ophthalmol..

[11] De Menthon M., LaValley M.P., Maldini C., Guillevin L., Mahr A. (2009). HLA-B51/B5and the risk of Behçet’s disease: A systematic review and meta-analysis of case-control genetic association studies. Arthritis Rheum..

[12] Kaklamani V.G., Vaiopoulos G., Kaklamanis P.G. (1998). Behçet’s Disease. Semin. Arthritis Rheum..

[13] Adil A., Goyal A., Bansal P., Quint J.M. (2020). Behcet Disease. StatPearls.

[14] Park J.H. (1999). Clinical Analysis of Behcet Disease; Arthritic Manifestations in Behcet Disease may present as Seronegative Rheumatoid Arthritis or Palindromic Rheumatism. Korean J. Intern. Med..

[15] Bicer A. (2011). Musculoskeletal Findings in Behcet’s Disease. Pathol. Res. Int..

[16] Yazici H., Tuzlaci M., Yurdakul S. (1981). A controlled survey of sacroiliitis in Behcet’s disease. Ann. Rheum. Dis..

[17] Hatemi G., Fresko I., Tascilar K., Yazici H. (2008). Increased enthesopathy among Behçet’s syndrome patients with acne and arthritis: An ultrasonography study. Arthritis Rheum..

[18] Dehlin M., Jacobsson L., Roddy E. (2020). Global epidemiology of gout: Prevalence, incidence, treatment patterns and risk factors. Nat. Rev. Rheumatol..

[19] Punzi L., Scanu A., Ramonda R., Oliviero F. (2012). Gout as autoinflammatory disease: New mechanisms for more appropriated treatment targets. Autoimmun. Rev..

[20] Shi Y., Evans J.E., Rock K.L. (2003). Molecular identification of a danger signal that alerts the immune system to dying cells. Nat. Cell Biol..

[21] Bjarnason I., O’Morain C., Levi A., Peters T.J. (1983). Absorption of 151chromium-labeled ethylenediaminetetraacetate in inflammatory bowel disease. Gastroenterology.

[22] McCarthy G.M., Dunne A. (2019). Calcium crystals and auto-inflammation. Rheumatology.

[23] Oliviero F., Bindoli S., Scanu A., Feist E., Doria A., Galozzi P., Sfriso P. (2020). Autoinflammatory Mechanisms in Crystal-Induced Arthritis. Front. Med..

[24] Richette P., Doherty M., Pascual E., Barskova V., Becce F., Castaneda J., Coyfish M., Guillo S., Jansen T., Janssens H. (2020). 2018 updated European League Against Rheumatism evidence-based recommendations for the diagnosis of gout. Ann. Rheum. Dis..

[25] Zhang W., Doherty M., Bardini T., Barskova V., Guerne P.-A., Jansen T.L., Leeb B.F., Perez-Ruiz F., Pimentao J., Punzi L. (2011). European League Against Rheumatism recommendations for calcium pyrophosphate deposition. Part I: Terminology and diagnosis. Ann. Rheum. Dis..

[26] Neame R.L., Carr A.J., Muir K., Doherty M. (2003). UK community prevalence of knee chondrocalcinosis: Evidence that correlation with osteoarthritis is through a shared association with osteophyte. Ann. Rheum. Dis..

[27] Felson D.T., Anderson J.J., Naimark A., Kannel W., Meenan R.F. (1989). The prevalence of chondrocalcinosis in the elderly and its association with knee osteoarthritis: The Framingham Study. J. Rheumatol..

[28] Salaffi F., De Angelis R., Grassi W., Prevalence M.P., INvestigation Group (MAPPING) study (2005). Prevalence of musculoskeletal conditions in an Italian population sample: Results of a regional community-based study. I. The MAPPING study. Clin. Exp. Rheumatol..

[29] Koto R., Nakajima A., Horiuchi H., Yamanaka H. (2020). Real-world treatment of gout and asymptomatic hyperuricemia: A cross-sectional study of Japanese health insurance claims data. Mod. Rheumatol..

[30] Abdellatif W., Ding J., Khorshed D., Shojania K., Nicolaou S. (2020). Unravelling the mysteries of gout by multimodality imaging. Semin. Arthritis Rheum..

[31] Checa A. (2019). Consistency of the sonographic image (double contour sign) in patients with gout after ambulation. J. Med. Ultrasound.

[32] Ogdie A., Taylor W., Neogi T., Fransen J., Jansen T.L., Schumacher H.R., Louthrenoo W., Vazquez-Mellado J., Eliseev M., McCarthy G. (2017). Performance of Ultrasound in the Diagnosis of Gout in a Multicenter Study: Comparison with Monosodium Urate Monohydrate Crystal Analysis as the Gold Standard. Arthritis Rheumatol..

[33] Ogdie A., Taylor W.J., Weatherall M., Fransen J., Jansen T.L., Neogi T., Schumacher H.R., Dalbeth N. (2015). Imaging modalities for the classification of gout: Systematic literature review and meta-analysis. Ann. Rheum. Dis..

[34] Peiteado D., Villalba A., Martín-Mola E., Balsa A., De Miguel E. (2017). Ultrasound sensitivity to changes in gout: A longitudinal study after two years of treatment. Clin. Exp. Rheumatol..

[35] Löffler C., Sattler H., Peters L., Tuleweit A., Löffler U., Wadsack D., Uppenkamp M., Bergner R. (2016). In arthritis the Doppler based degree of hypervascularisation shows a positive correlation with synovial leukocyte count and distinguishes joints with leukocytes greater and less than 5/nL. Jt. Bone Spine.

[36] Lee Y.H., Song G.G. (2017). Diagnostic accuracy of dual-energy computed tomography in patients with gout: A meta-analysis. Semin. Arthritis Rheum..

[37] Gamala M., Jacobs J.W.G., Van Laar J.M. (2019). The diagnostic performance of dual energy CT for diagnosing gout: A systematic literature review and meta-analysis. Rheumatology.

[38] Bongartz T., Glazebrook K.N., Kavros S.J., Murthy N.S., Merry S.P., Franz W.B., Michet C.J., Veetil B.M.A., Davis J.M.I., Ii T.G.M. (2015). Dual-energy CT for the diagnosis of gout: An accuracy and diagnostic yield study. Ann. Rheum. Dis..

[39] Kotlyarov M., Hermann K.G.A., Mews J., Hamm B., Diekhoff T. (2020). Development and validation of a quantitative method for estimation of the urate burden in patients with gouty arthritis using dual-energy computed tomography. Eur. Radiol..

[40] Dalbeth N., Choi H.K. (2013). Dual-Energy Computed Tomography for Gout Diagnosis and Management. Curr. Rheumatol. Rep..

[41] Diekhoff T., Scheel M., Hermann S., Mews J., Hamm B., Hermann K.-G.A. (2017). Osteitis: A retrospective feasibility study comparing single-source dual-energy CT to MRI in selected patients with suspected acute gout. Skelet. Radiol..

[42] Filippou G., Scirè C.A., Adinolfi A., Damjanov N.S., Carrara G., Bruyn G.A.W., Cazenave T., D’Agostino M.A., Sedie A.D., Di Sabatino V. (2018). Identification of calcium pyrophosphate deposition disease (CPPD) by ultrasound: Reliability of the OMERACT definitions in an extended set of joints—An international multiobserver study by the OMERACT Calcium Pyrophosphate Deposition Disease Ultrasound Subtask Force. Ann. Rheum. Dis..

[43] Filippou G., Scirè C.A., Damjanov N., Adinolfi A., Carrara G., Picerno V., Toscano C., Bruyn G.A., D’Agostino M.A., Sedie A.D. (2017). Definition and Reliability Assessment of Elementary Ultrasonographic Findings in Calcium Pyrophosphate Deposition Disease: A Study by the OMERACT Calcium Pyrophosphate Deposition Disease Ultrasound Subtask Force. J. Rheumatol..

[44] Chang E.Y., Lim W.Y., Wolfson T., Gamst A., Chung C.B., Bae W.C., Resnick D.L. (2013). Frequency of Atlantoaxial Calcium Pyrophosphate Dihydrate Deposition at CT. Radiology.

[45] Ziegeler K., Diekhoff T., Hermann S., Hamm B., Hermann K.G. (2019). Low-dose computed tomography as diagnostic tool in calcium pyrophosphate deposition disease arthropathy: Focus on ligamentous calcifications of the wrist. Clin. Exp. Rheumatol..

[46] Misra D., Guermazi A., Sieren J.P., Lynch J.A., Torner J.C., Neogi T., Felson D. (2015). CT imaging for evaluation of calcium crystal deposition in the knee: Initial experience from the Multicenter Osteoarthritis (MOST) study. Osteoarthr. Cartil..

[47] Diekhoff T., Kiefer T., Stroux A., Pilhofer I., Juran R., Mews J., Blobel J., Tsuyuki M., Ackermann B., Hamm B. (2015). Detection and Characterization of Crystal Suspensions Using Single-Source Dual-Energy Computed Tomography. Investig. Radiol..

[48] Pascart T., Falgayrac G., Norberciak L., Lalanne C., Legrand J., Houvenagel E., Ea H.-K., Becce F., Budzik J.-F. (2020). Dual-energy computed-tomography-based discrimination between basic calcium phosphate and calcium pyrophosphate crystal deposition in vivo. Ther. Adv. Musculoskelet. Dis..

[49] Pascart T., Norberciak L., Legrand J., Becce F., Budzik J. (2019). Dual-energy computed tomography in calcium pyrophosphate deposition: Initial clinical experience. Osteoarthr. Cartil..

[50] Filippou G., Pascart T., Iagnocco A. (2020). Utility of Ultrasound and Dual Energy CT in Crystal Disease Diagnosis and Management. Curr. Rheumatol. Rep..

[51] Ziegeler K., Hermann S., Hermann K.G.A., Hamm B., Diekhoff T. (2019). Dual-energy CT in the differentiation of crystal depositions of the wrist: Does it have added value?. Skelet. Radiol..

[52] Ziegeler K., Richter S.-T., Hermann S., Hermann K.G.A., Hamm B., Diekhoff T. (2020). Dual-energy CT collagen density mapping of wrist ligaments reveals tissue remodeling in CPPD patients: First results from a clinical cohort. Skelet. Radiol..

[53] Moshrif A., Laredo J.D., Bassiouni H., Abdelkareem M., Richette P., Rigon M.R., Bardin T. (2019). Spinal involvement with calcium pyrophosphate deposition disease in an academic rheumatology center: A series of 37 patients. Semin. Arthritis Rheum..

[54] Hongsmatip P., Cheng K.Y., Kim C., Lawrence D.A., Rivera R., Smitaman E. (2019). Calcium hydroxyapatite deposition disease: Imaging features and presentations mimicking other pathologies. Eur. J. Radiol..

[55] Chianca V., Albano D., Messina C., Midiri F., Mauri G., Aliprandi A., Catapano M., Pescatori L.C., Monaco C.G., Gitto S. (2018). Rotator cuff calcific tendinopathy: From diagnosis to treatment. Acta Biomed..

[56] Jacques T., Michelin P., Badr S., Nasuto M., Lefebvre G., Larkman N., Cotten A. (2017). Conventional Radiology in Crystal Arthritis. Radiol. Clin. N. Am..

[57] Cho N.S., Lee B.G., Rhee Y.G. (2010). Radiologic course of the calcific deposits in calcific tendinitis of the shoulder: Does the initial radiologic aspect affect the final results?. J. Shoulder Elb. Surg..

[58] Uhthoff H., Sarkar K. (1989). Calcifying tendinitis. Baillières Clin. Rheumatol..

[59] Greis A.C., Derrington S.M., McAuliffe M. (2015). Evaluation and Nonsurgical Management of Rotator Cuff Calcific Tendinopathy. Orthop. Clin. N. Am..

[60] Ea H., Lioté F. (2004). Calcium pyrophosphate dihydrate and basic calcium phosphate crystalinduced arthropathies: Update on pathogenesis, clinical features, and Therapy. Curr. Rheumatol. Rep..

[61] McCarty D.J. (1991). Milwaukee shoulder syndrome. Trans. Am. Clin. Climatol. Assoc..

[62] Santiago T., Coutinho M., Malcata A., Da Silva J.A.P. (2014). Milwaukee shoulder (and knee) syndrome. BMJ Case Rep..

[63] Feist E., Mitrovic S., Fautrel B. (2018). Mechanisms, biomarkers and targets for adult-onset Still’s disease. Nat. Rev. Rheumatol..

[64] Fautrel B. (2008). Adult-onset Still disease. Best Pract. Res. Clin. Rheumatol..

[65] Pouchot J., Sampalis J.S., Beaudet F., Carette S., Decary F., Salusinsky-Sternbach M., Hill R.O., Gutkowski A., Harth M., Myhal D. (1991). Adult Still’s disease: Manifestations, disease course, and outcome in 62 patients. Medicine.

[66] Belghali S., El Amri N., Baccouche K., Laataoui S., Bouzaoueche M., Zeglaoui H., Bouajina E. (2018). Atypical form of Adult-onset Still’s Disease with Distal Interphalangeal Joints Involvement. Curr. Rheumatol. Rev..

[67] Jelušić M., Čekada N., Frković M., Potočki K., Skerlev M., Murat-Sušić S., Husar K., Đapić T., Šmigovec I., Bajramović D. (2018). Chronic Recurrent Multifocal Osteomyelitis (CRMO) and Synovitis Acne Pustulosis Hyperostosis Osteitis (SAPHO) Syndrome—Two Presentations of the Same Disease?. Acta Dermatovenerol. Croat..

[68] Jansson M.A., Renner E.D., Ramser J., Mayer A., Haban M., Meindl A., Grote V., Diebold J., Schneider K., Belohradsky B.H. (2007). Classification of Non-Bacterial Osteitis: Retrospective study of clinical, immunological and genetic aspects in 89 patients. Rheumatology.

[69] Klicman R.F., Simoni P., Robinson P., Teh J., Jurik A.G. (2018). SAPHO and CRMO: The Value of Imaging. Semin. Musculoskelet. Radiol..

[70] Falip C., Alison M., Boutry N., Job-Deslandre C., Cotten A., Azoulay R., Adamsbaum C. (2013). Chronic recurrent multifocal osteomyelitis (CRMO): A longitudinal case series review. Pediatr. Radiol..

[71] Jurik A.G. (2004). Chronic Recurrent Multifocal Osteomyelitis. Semin. Musculoskelet. Radiol..

[72] DePasquale R., Kumar N., Lalam R., Tins B., Tyrrell P., Singh J., Cassar-Pullicino V. (2012). SAPHO: What radiologists should know. Clin. Radiol..

[73] Andronikou S., Kraft J.K., Offiah A.C., Jones J., Douis H., Thyagarajan M., Barrera C.A., Zouvani A., Ramanan A.V. (2020). Whole-body MRI in the diagnosis of paediatric CNO/CRMO. Rheumatology.

[74] Buch K., Thuesen A.C.B., Brøns C., Schwarz P. (2019). Chronic Non-bacterial Osteomyelitis: A Review. Calcif. Tissue Int..

[75] Chimenti M.S., Caso F., Alivernini S., De Martino E., Costa L., Tolusso B., Triggianese P., Conigliaro P., Gremese E., Scarpa R. (2019). Amplifying the concept of psoriatic arthritis: The role of autoimmunity in systemic psoriatic disease. Autoimmun. Rev..

[76] Himuro H., Kurata S., Nagata S., Sumi A., Tsubaki F., Matsuda A., Fujimoto K., Abe T. (2020). Imaging features in patients with SAPHO/CRMO: A pictorial review. Jpn. J. Radiol..

